# Supplementary material for: Regulation of bone homeostasis by MERTK and TYRO3
Source: Nat Commun. 2022 Dec 12;13:7689. doi: 10.1038/s41467-022-33938-x (PMC9744875; doi:10.1038/s41467-022-33938-x)
Supplement: Supplementary file 1 — Supplementary Information [file 41467_2022_33938_MOESM1_ESM.pdf]

Supplementary Figure 1: Conditional knockout of *Tyro3.*

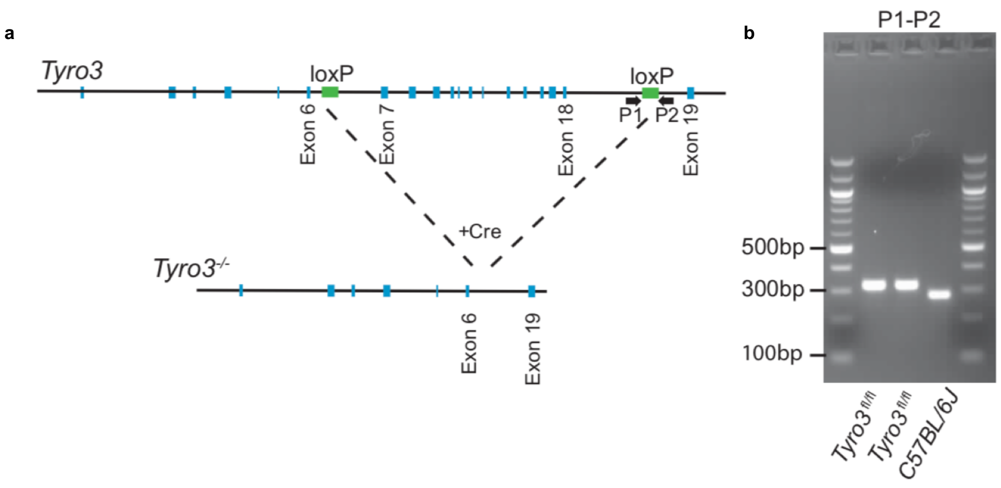

a, Structure of the mouse *Tyro3* gene, indicating the loxP sites (top) and the recombined allele (bottom). Recombination leads to excision of exons 7 through 18, as indicated. **b,** PCR amplification of gDNA from Tyro3$^{flox/flox}$ using primers P1-P2. Tyro3$^{flox/flox}$ (321bp band), wild-type C57BL/6J (281bp band).

Supplementary Figure 2: **Body weight of *Col1a1-cre*$^{+}$*;Mertk*$^{flox/flox}$ and *Col1a1-cre*$^{+}$*;Tyro3*$^{flox/flox}$ mice.**

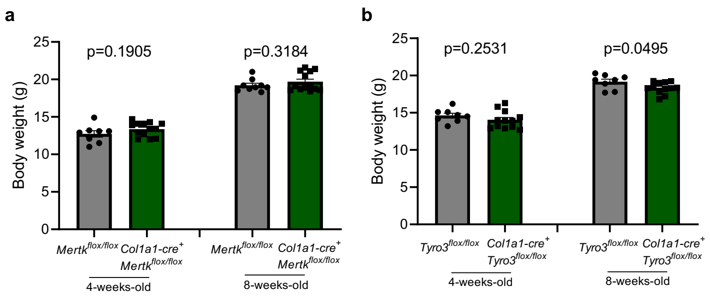

a, b, Body weight of 4- and 8-week-old *Mertk*$^{flox/flox}$ and *Col1a1-cre*$^{+}$*;Mertk*$^{flox/flox}$ mice (n=8/13) (**a**) and *Tyro3*$^{flox/flox}$ and *Col1a1-cre*$^{+}$*;Tyro3*$^{flox/flox}$ mice (n=8/11) (**b**). Data are means ± SEMs. Statistical significance was determined by two-tailed unpaired t-test.

Supplementary Figure 3: Target gene expression in osteoblast calvarial cell cultures of *Col1a1-cre*$^{+}$*;Mertk*$^{flox/flox}$ and *Col1a1-cre*$^{+}$*;Tyro3*$^{flox/flox}$ mice.

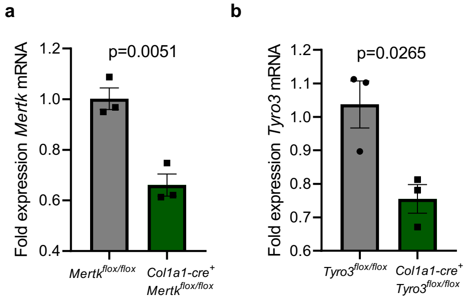

a, b, RT-qPCR analysis of *Mertk* and *Tyro3* mRNA expression in ex vivo calvarial cell cultures of *Col1a1-cre*$^{+}$*;Mertk*$^{flox/flox}$ **(a)** and *Col1a1-cre*$^{+}$*;Tyro3*$^{flox/flox}$ **(b)** mice on day 5 after osteogenic induction (n=3 biological replicates). Data are means ± SEMs. Statistical significance was determined by two-tailed unpaired t-test.

Supplementary Figure 4: **μCT analysis of *Col1a1-cre*⁺*;Mertk*$^{flox/flox}$ mice.**

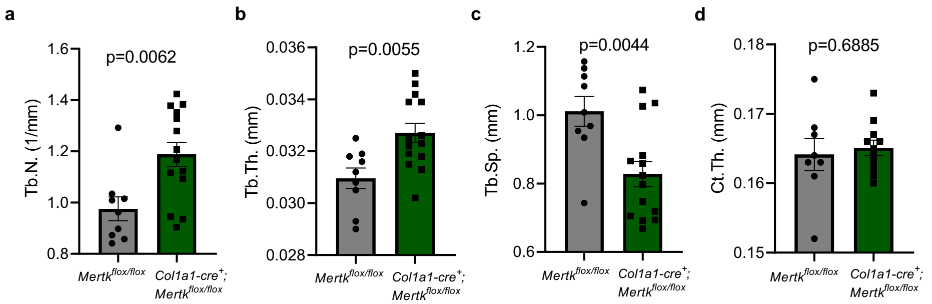

Microcomputed tomography (μCT) of the metaphyseal proximal region of tibias from 8-week-old *Mertk*$^{flox/flox}$ and *Col1a1-cre*$^{+}$*;Mertk*$^{flox/flox}$ female mice. **a-c,** Quantification of trabecular number **(a)**, trabecular thickness **(b)** and trabecular separation **(c)** of cancellous bone determined by μCT analysis (*Mertk*$^{flox/flox}$, n=9; *Col1a1-cre*$^{+}$*;Mertk*$^{flox/flox}$, n=14). **d,** Midshaft evaluation of cortical bone of femur of 8-week-old *Mertk*$^{flox/flox}$ and *Col1a1-cre*$^{+}$*;Mertk*$^{flox/flox}$ female mice (*Mertk*$^{flox/flox}$, n=8; *Col1a1-cre*$^{+}$*;Mertk*$^{flox/flox}$, n=11). Data are means ± SEMs. Statistical significance was determined by two-tailed unpaired t-test.

Supplementary Figure 5: Genetic deletion of *Mertk* reduces migration and focal adhesion formation in osteoblasts.

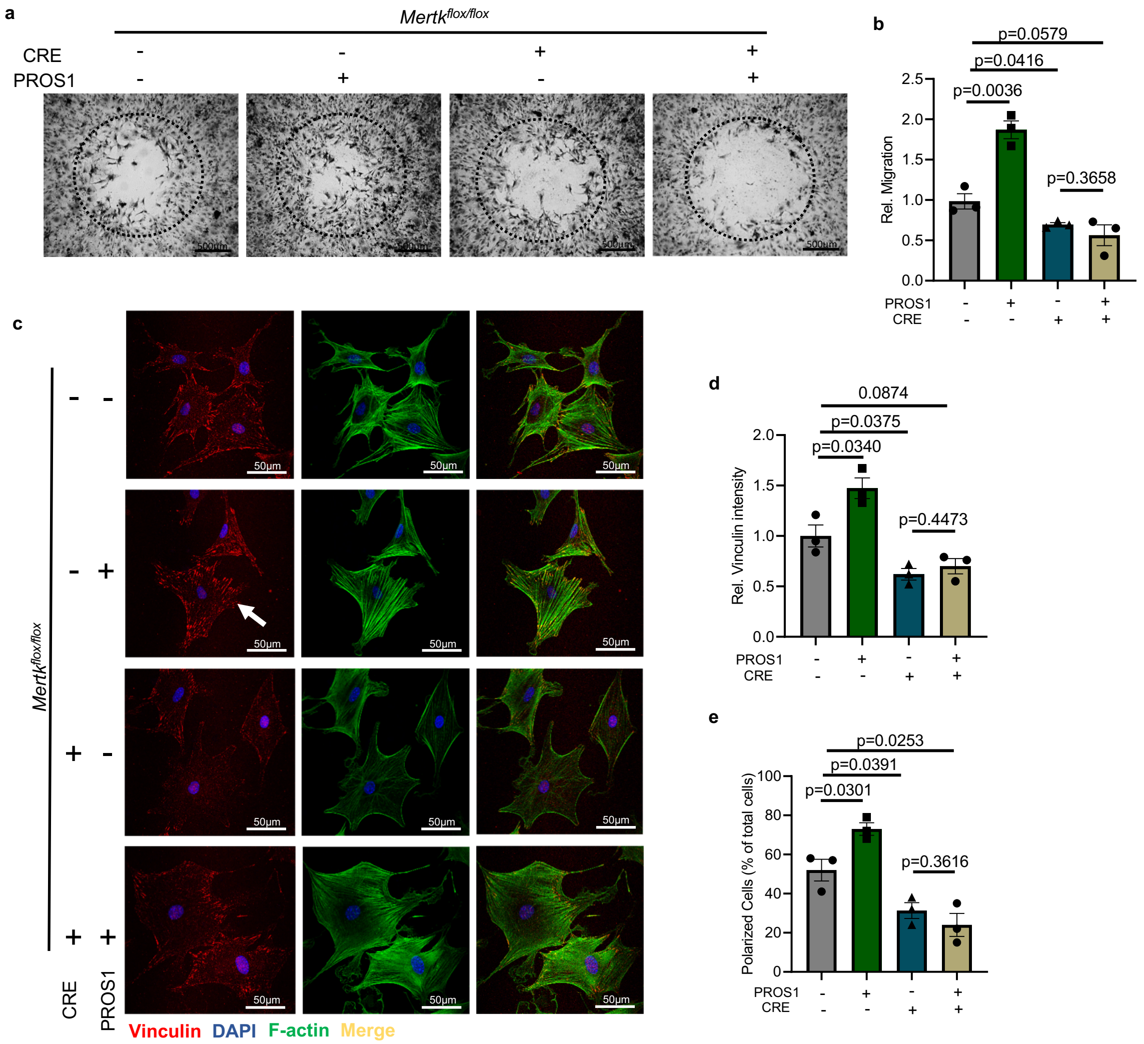

a, b, Wound healing migration assay of osteoblasts lacking *Mertk.* Representative pictures **(a)** and quantification of relative migration (n=3 biological replicates) **(b)**. **c,** Detection of focal adhesion formation by vinculin immunofluorescence staining in osteoblasts lacking *Mertk* and treated with PROS1. White arrow pointing to vinculin-rich focal adhesions. **d,** Vinculin staining intensity was quantified (n=3, mean of 50 measurements in 3 fields). **e,** Cells with a leading and a trailing edge were quantified (n=3, mean of 100 cells in 3 fields). Data are means ± SEMs. Statistical significance was determined by two-tailed unpaired t-test.

Supplementary Figure 6: **µCT analysis of *Col1a1-cre*$^{+}$*;Tyro3*$^{flox/flox}$ mice.**

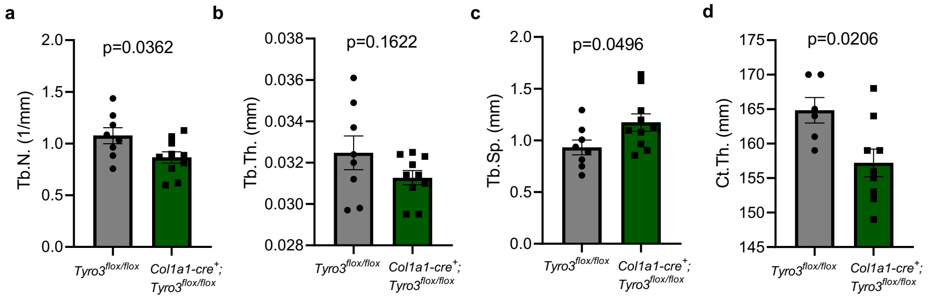

a-d, Microcomputed tomography (µCT) of the metaphyseal proximal region of tibias from 8-week-old *Tyro3*$^{flox/flox}$ and *Col1a1-cre*$^{+}$*;Tyro3*$^{flox/flox}$ female mice. Quantification of trabecular number **(a)**, trabecular thickness **(b)** and trabecular separation **(c)** of cancellous bone determined by µCT analysis (*Tyro3*$^{flox/flox}$, n=8; *Col1a1-cre*$^{+}$*;Tyro3*$^{flox/flox}$, n=10). **d,** Midshaft evaluation of cortical bone of femur of 8-week-old *Tyro3*$^{flox/flox}$ and *Col1a1-cre*$^{+}$*;Tyro3*$^{flox/flox}$ female mice (*Tyro3*$^{flox/flox}$, n=6; *Col1a1-cre*$^{+}$*;Tyro3*$^{flox/flox}$, n=*9*). Data are means ± SEMs. Statistical significance was determined by two-tailed unpaired t-test.

Supplementary Figure 7: Genetic deletion of *Tyro3* increases migration and focal adhesion formation in osteoblasts.

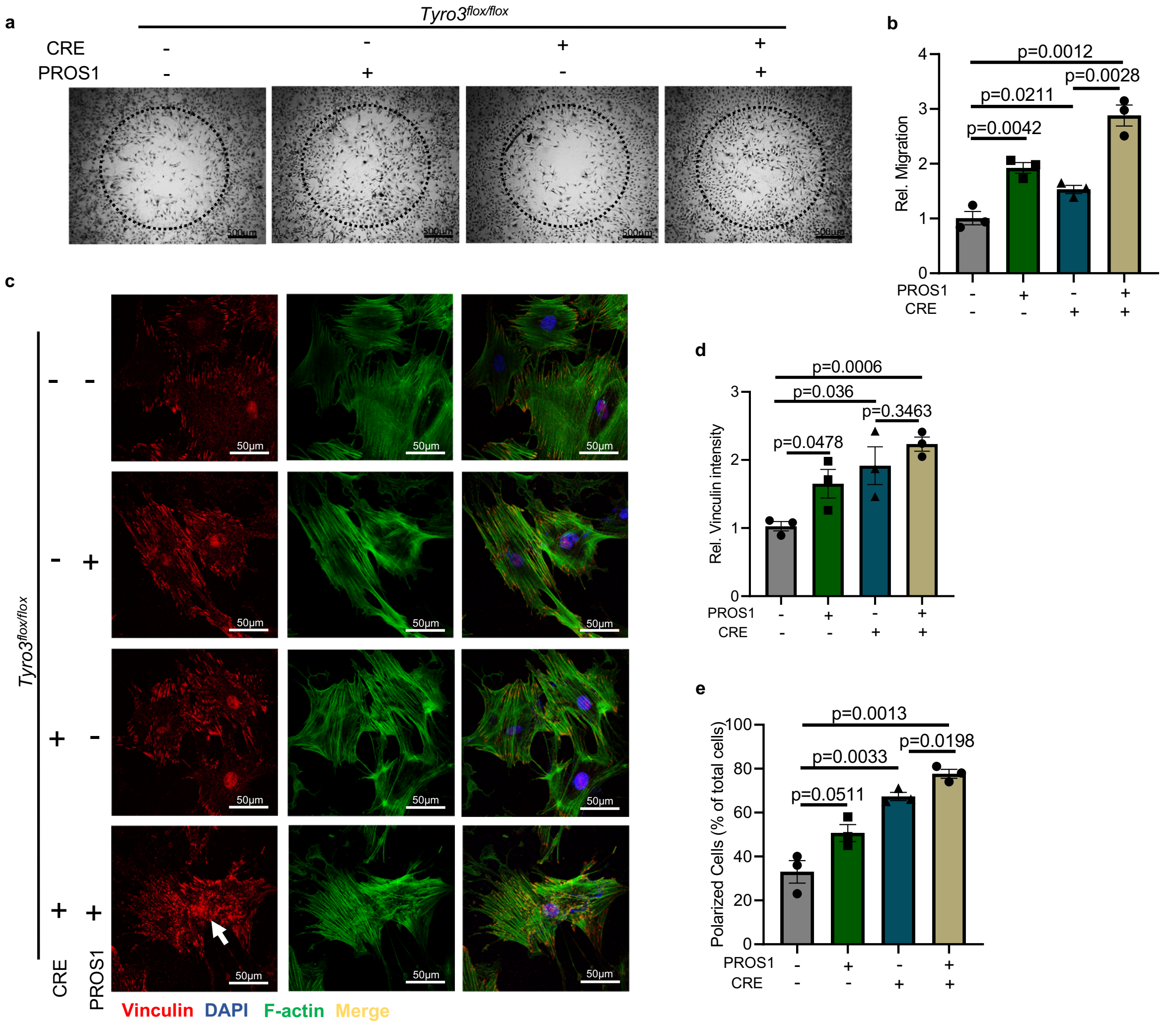

a, b, Wound healing migration assay of osteoblasts lacking *Tyro3.* Representative pictures **(a)** and quantification of relative migration **(b)** (n=3 biological replicates). **c,** Detection of focal adhesion formation by vinculin immunofluorescence staining in osteoblasts lacking *Tyro3* and treated with PROS1. White arrow pointing to vinculin-rich focal adhesions. **d,** Vinculin staining intensity was quantified (n=3, mean of 50 measurements in 3 fields). **e,** Cells with a leading and a trailing edge were quantified (n=3, mean of 100 cells in 3 fields). Data are means ± SEMs. Statistical significance was determined by two-tailed unpaired t-test.

Supplementary Figure 8: siRNA-mediated knockdown of *Mertk* and *Tyro3* in osteoblast calvarial cell cultures.

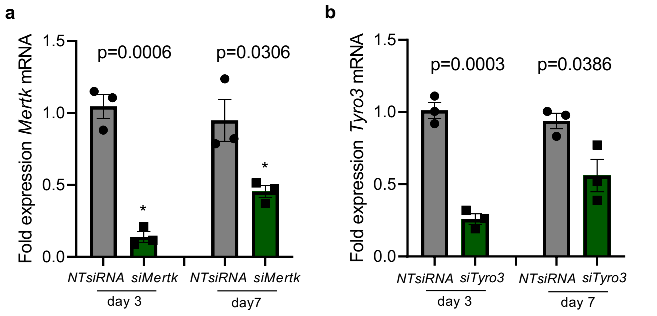

a, b, RT-qPCR analysis of *Mertk* and *Tyro3* mRNA expression of siRNA mediated knockdown of *Mertk* (**a**) and *Tyro3* (**b**) in calvarial cell cultures (n=3 biological replicates). Data are means ± SEMs. Statistical significance was determined by two-tailed unpaired t-test.

Supplementary Figure 9: *Mertk* and *Tyro3* control the F-actin cytoskeleton in osteoblasts.

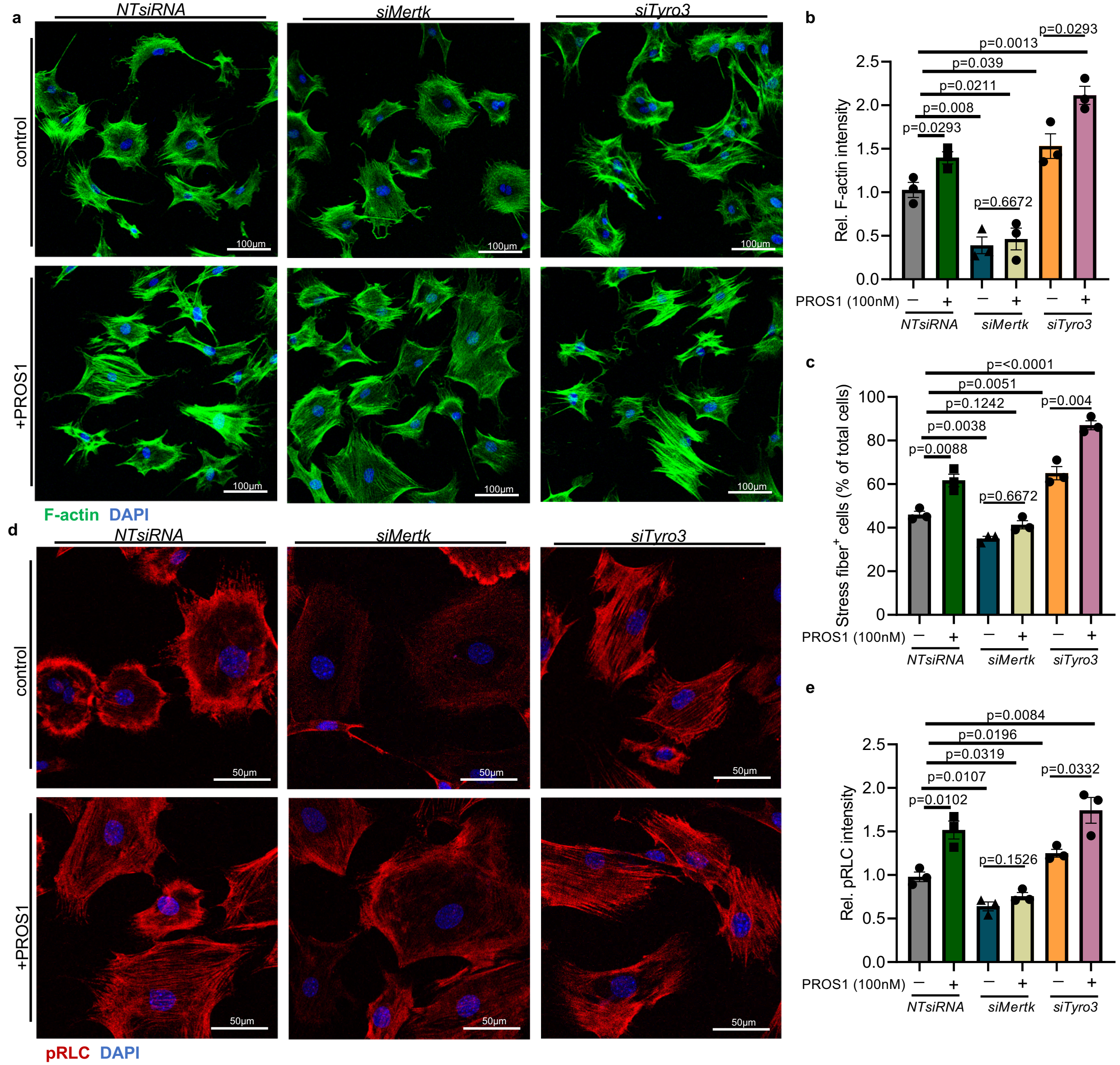

a, Confocal imaging of F-actin staining of osteoblast cultures and osteoblast single cell analysis silenced for *Mertk* and *Tyro3* and treated with TAM receptor ligand PROS1 (100nM) on glass cover slips. **b,** F-actin immunofluorescence intensity of *Mertk* and *Tyro3* silenced osteoblasts treated with PROS1 (n=3, mean of 50 measurements in 3 fields). **c,** Quantification of stress fiber containing cells (n=3, mean of 100 cells in 3 fields). **d,** Representative confocal images of pRLC staining in *Mertk* and *Tyro3* silenced osteoblasts treated with PROS1. **e,** pRLC immunofluorescence intensity (n=3, mean of 50 measurements in 3 fields). Data are means ± SEMs. Statistical significance was determined by two-tailed unpaired t-test.

Supplementary Figure 10: ***Mertk* and *Tyro3* conversely regulate migration and focal adhesion formation in osteoblasts.**

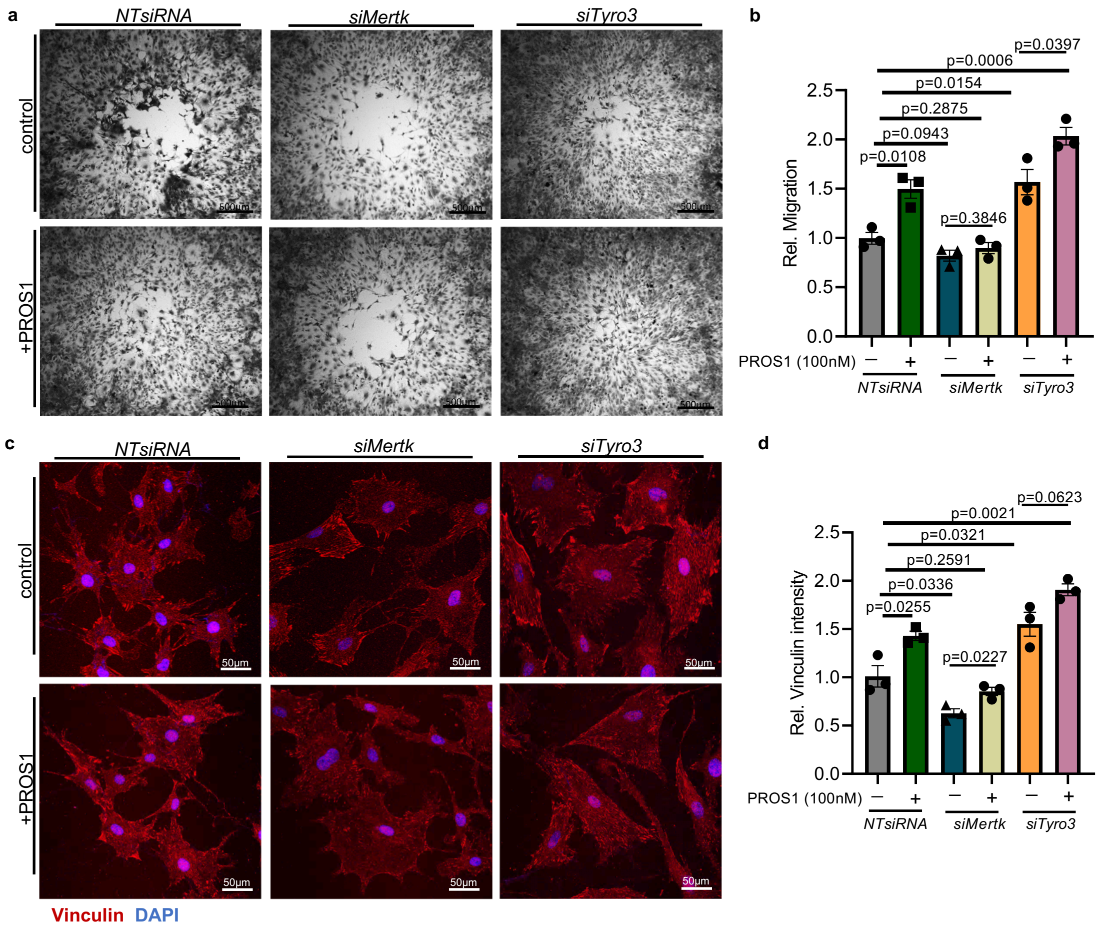

a,b, Wound healing migration assay of osteoblasts lacking *Mertk* and *Tyro3* and treated with PROS1 (100nM). Representative pictures **(a)** and quantification of the relative migration **(b)** (n=3 biological replicates). **c,** Detection of focal adhesion formation by vinculin immunofluorescence staining in osteoblasts lacking *Mertk* and *Tyro3* and treated with PROS1 (100nM). **d,** Vinculin staining intensity was quantified (n=3, mean of 50 measurements in 3 fields). Data are means ± SEMs. Statistical significance was determined by two-tailed unpaired t-test.

Supplementary Figure 11: ***Mertk* and *Tyro3* conversely regulate cell polarization and spreading in osteoblasts**

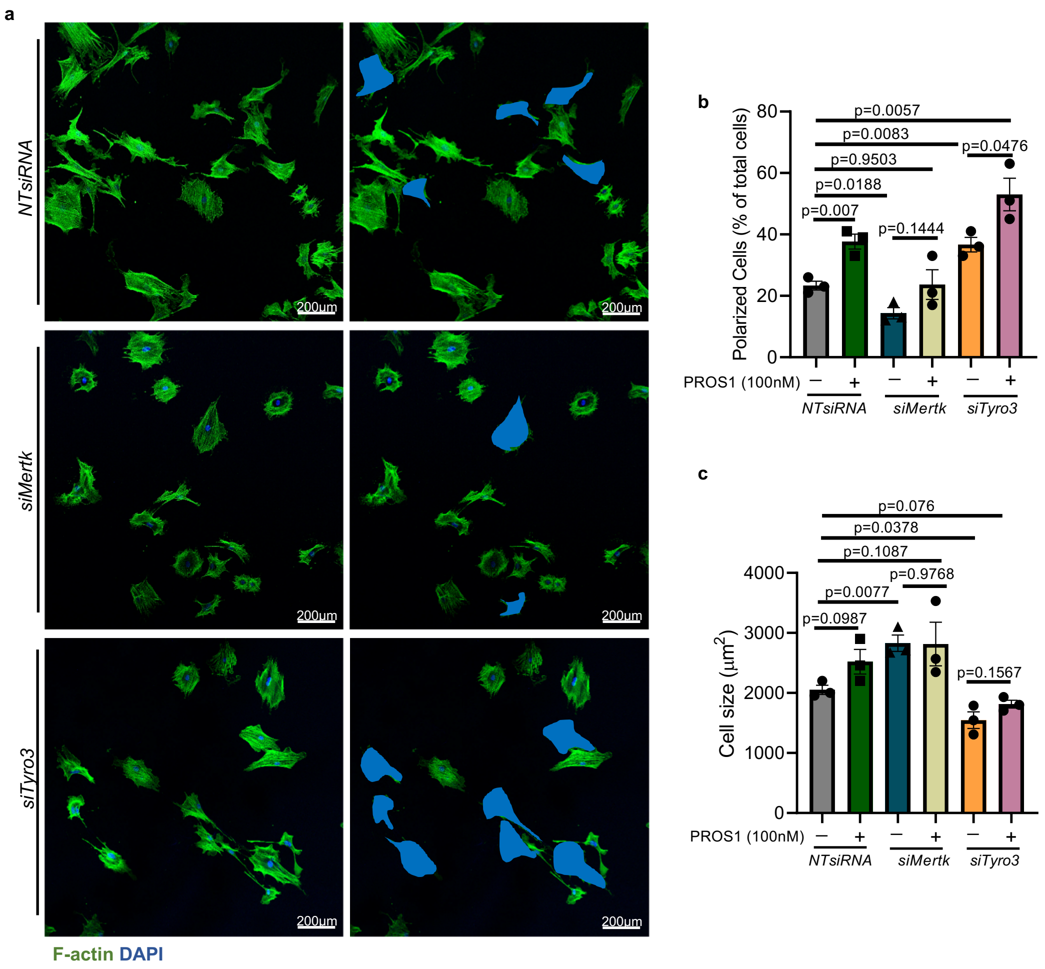

a, Confocal images (10x objective) of F-actin staining of *Mertk*- and *Tyro3*-silenced osteoblasts. Cells exhibiting a leading and a trailing edge are marked in blue. **b,** Cells with a leading and a trailing edge were quantified in presence and absence of PROS1 (n=3, mean of 100 cells in 3 fields). **c,** Quantification of cell area in a spreading assay after 20 min (n=mean of 50-100 measurements in 3 random fields). Data are means ± SEMs. Statistical significance was determined by two-tailed unpaired t-test.

Supplementary Figure 12: ***Mertk* and *Tyro3* conversely regulate RHOA activity in osteoblasts.**

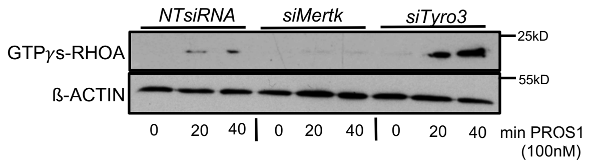

Immunoblot of activated GTPγs-bound RHOA in *Mertk* and *Tyro3* silenced osteoblasts treated with TAM receptor ligand PROS1. ß-ACTIN run on a separate gel.

Supplementary Figure 13: R992 counteracts cancer cell-mediated inhibition of osteoblastic bone formation and reduces osteoblast motility.

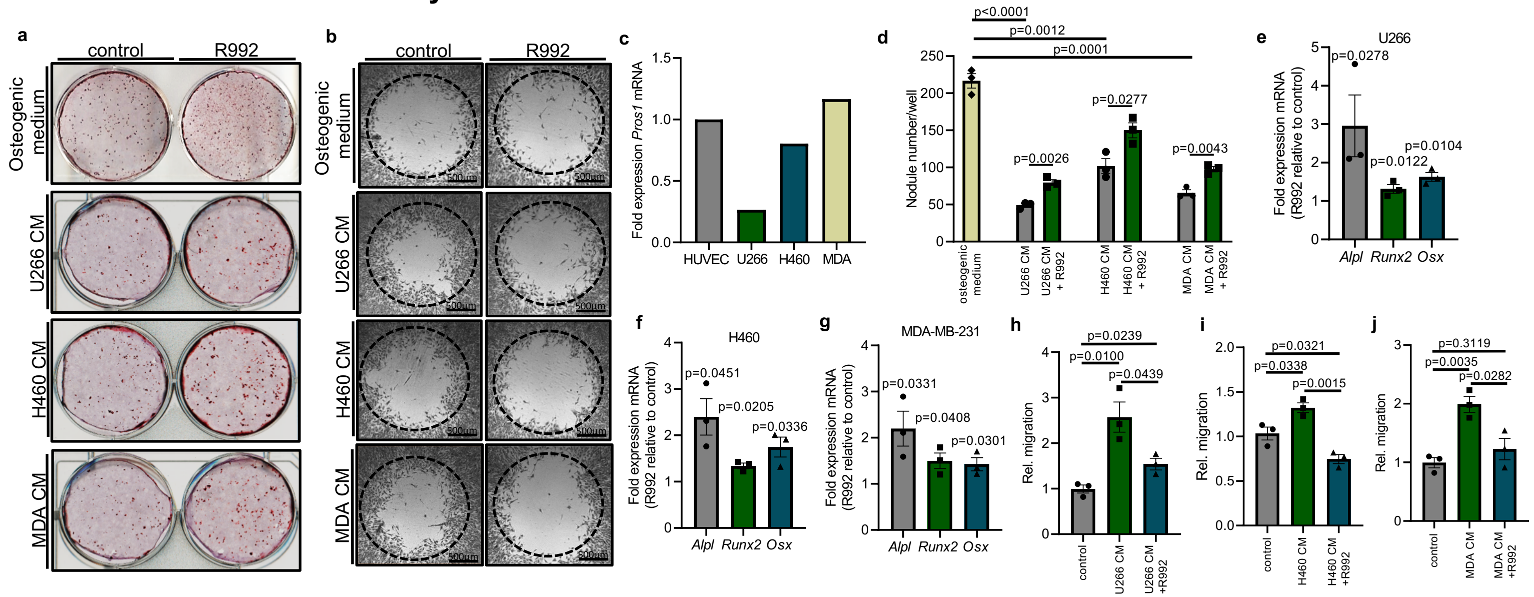

a, b, Photographs of Alizarin Red staining **(a)** and wound healing migration assays **(b)** of osteoblast cultures incubated with osteogenic medium or tumor conditioned medium (CM) of U266, H460 and MDA-MB-231 cancer cells and with +/- R992. **c,** *Pros1* mRNA expression in U266, H460 and MDA-MB-231 cells normalized to human umbilical vein endothelial cells (HUVEC). Endothelial cells represent a major source of PROS1 (n=2 technical replicates). **d,** Quantification of bone nodule formation in osteoblast cultures incubated with conditioned medium from U266, H460 and MDA-MB-231 cells and treated with R992 (n=3 biological replicates). **e-g,** *Alpl*, *Runx2*, and *SP7* mRNA expression in osteoblasts in the presence of tumor CM from U266 **(e)**, H460 **(f)** or MDA-MB-231 **(g)** and R992 compared to osteoblasts incubated with tumor CM alone (n=3 biological replicates). **h-j,** Analysis of osteoblast migration induced by tumor CM from U266 **(h)**, H460 **(i)** or MDA-MB-231 **(j)** and R992 treatment in relation to osteoblasts incubated with tumor CM alone (n=3 biological replicates). Data are means ± SEMs. Statistical significance was determined by two-tailed unpaired t-test.

Supplementary Figure 14: Tumor cells induce a MERTK-dependent cytoskeletal reorganization in osteoblasts.

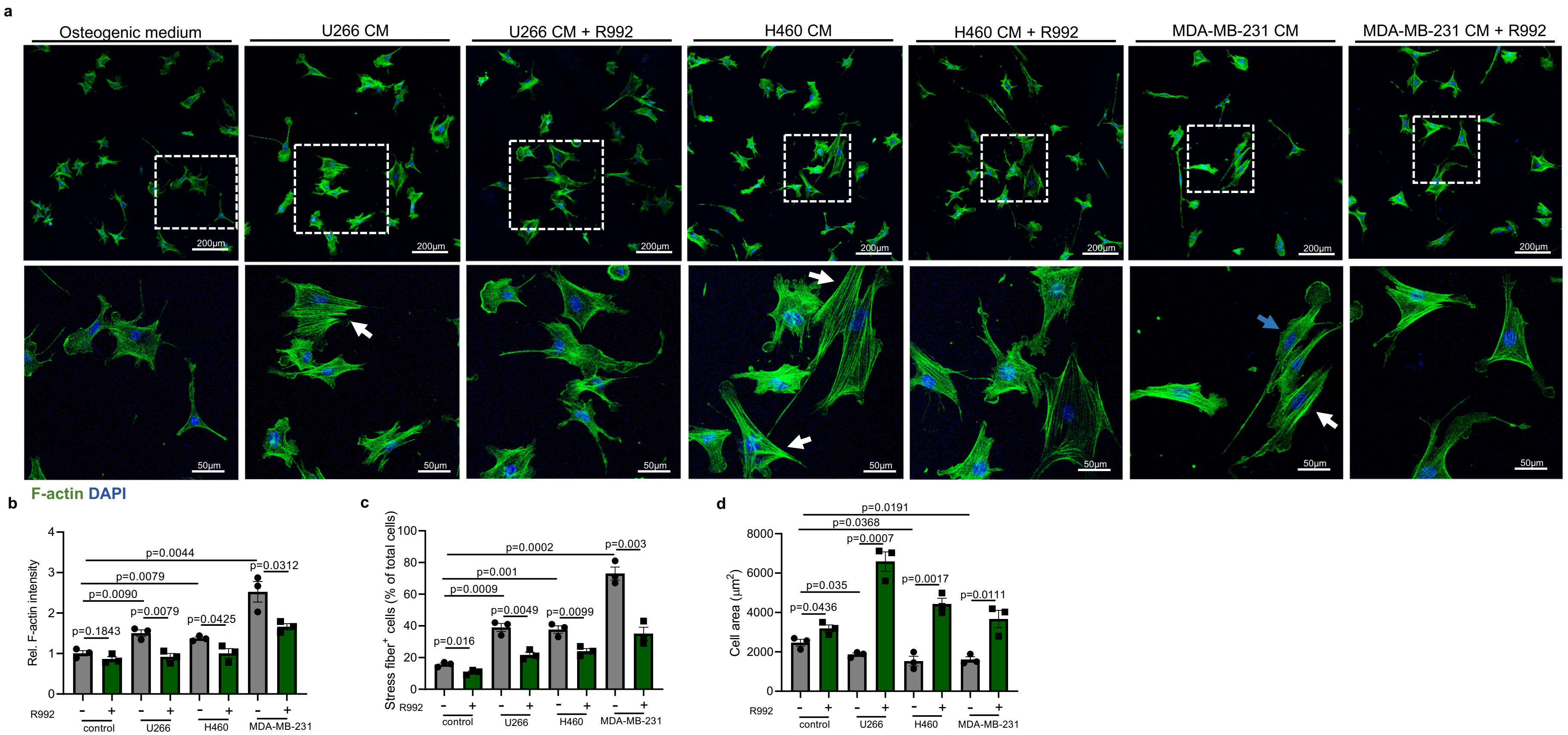

a, Confocal imaging (10x and 40x objective) of F-actin staining of osteoblasts on fibronectin coated glass cover slips. Osteoblasts were incubated with osteogenic medium (control) or tumor conditioned medium (CM) of U266, H460 and MDA-MB-231 cells +/- R992 for 48h. **b,** F-actin immunofluorescence staining intensity was quantified (n=50 measurements in 3 fields). **c,** Stress fibers induced by tumor CM +/- R992 were quantified (n=100 measurements in 3 fields). **d,** For cell spreading assays osteoblasts cultured with tumor CM were seeded out +/- R992 on fibronectin for 20 min and the cell area was quantified (n=3, mean of 50 measurements in 3 fields). Data are means ± SEMs. Statistical significance was determined by two-tailed unpaired t-test.

Supplementary Fig. 15: MERTK blockade inhibits tumor progression in the RPMI8226 Multiple Myeloma mouse model.

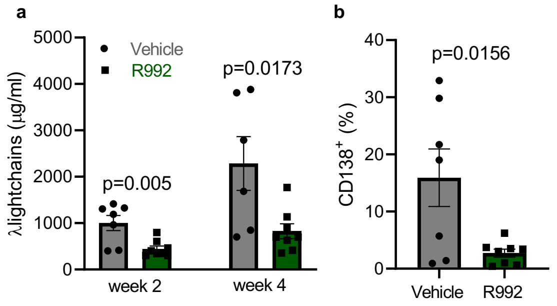

a,b, Blockade of MERTK by R992 (60mg/kg BID) in NSG mice injected with RPMI8226 cells reduces human Igλ light chain paraprotein in the peripheral blood after 2 (n=7/8) and 4 (n=6/8) weeks **(a)** and myeloma cell infiltration after 4 weeks (n=7/8) **(b)**. Data are means ± SEMs. Statistical significance was determined by two-tailed unpaired t-test.

Supplementary Fig. 16: µCT analysis U266 mouse model.

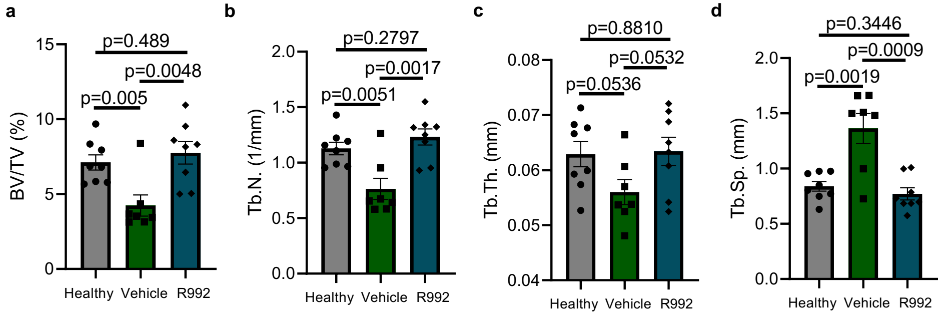

a-d, Microcomputed tomography (µCT) analysis of bone volume/total volume (BV/TV) **(a)**, trabecular number (Tb.N.) **(b)**, trabecular thickness (Tb.Th.) **(c)** and trabecular separation (Tb.Sp.) **(d)** of the metaphyseal proximal region of femur in the U266 mouse model in comparison to healthy age- and sex matched controls (Healthy n=8, Vehicle n=7, R992 n=8). Data are means ± SEMs. Statistical significance was determined by two-tailed unpaired t-test.

Supplementary Fig. 17: MERTK blockade by R992 inhibits tumor progression in metastasis mouse models of breast- and lung cancer.

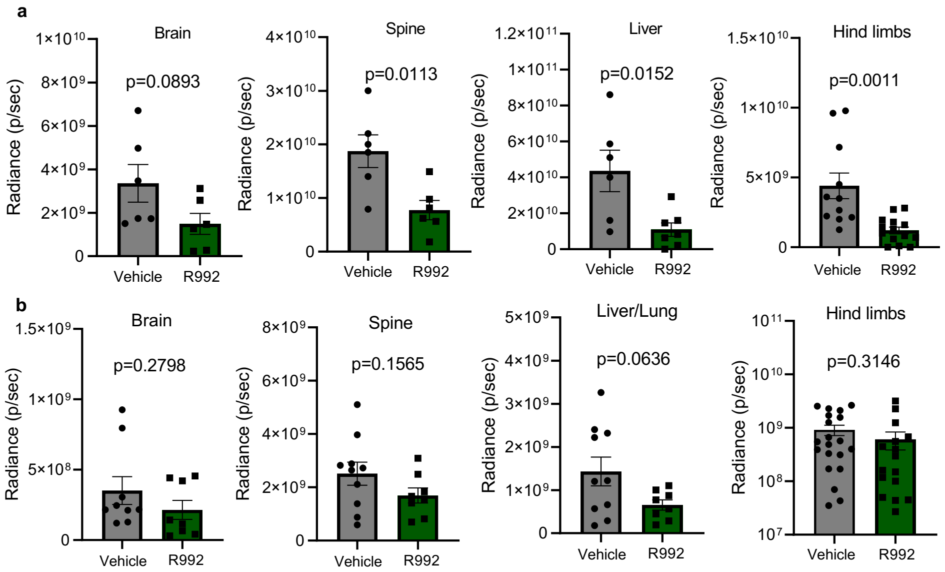

a, Analysis of radiance intensity of the H460 mouse model in the brain (n=6/6), spine (n=6/6), liver (n=6/7) and hind limbs (n=11/14). **b,** Analysis of radiance intensity of the MDA-MB-231 mouse model in the brain (n=9/8), spine (n=10/8), liver (n=10/8) and hind limbs (n=20/16). Data are means ± SEMs. Statistical significance was determined by two-tailed unpaired t-test.

Supplementary Fig. 18: µCT analysis H460 mouse model.

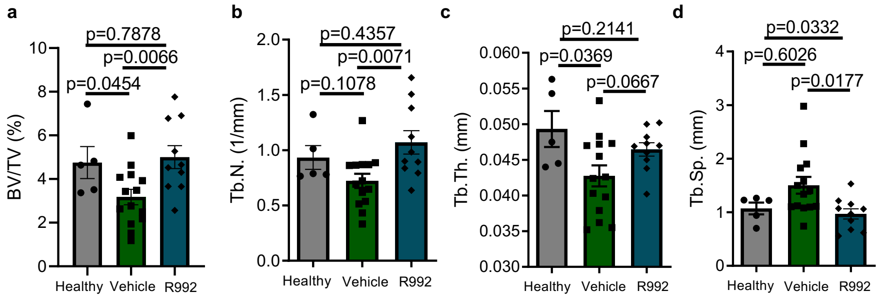

a-d, Microcomputed tomography (µCT) analysis of bone volume/total volume (BV/TV) **(a)**, trabecular number (Tb.N.) **(b)**, trabecular thickness (Tb.Th.) **(c)** and trabecular separation (Tb.Sp.) **(d)** of the metaphyseal proximal region of tibias in the H460 mouse model in comparison to healthy age-and sex matched controls (Healthy n=5, Vehicle n=14, R992 n=10). Data are means ± SEMs. Statistical significance was determined by two-tailed unpaired t-test.

Supplementary Fig. 19: µCT analysis MDA-MB-231 mouse model.

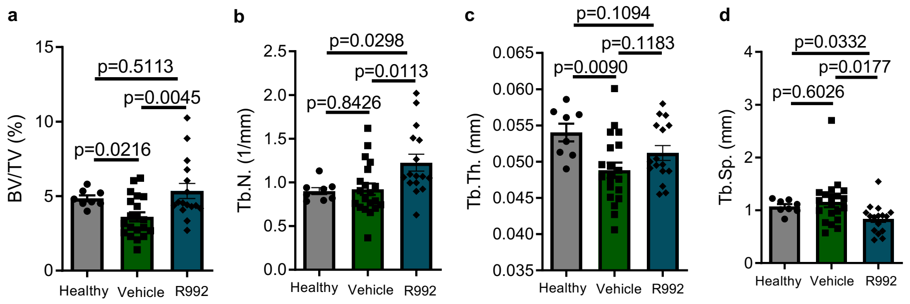

a-d, Microcomputed tomography (µCT) analysis of bone volume/total volume (BV/TV) (**a**), trabecular number (Tb.N.) (**b**), trabecular thickness (Tb.Th.) (**c**) and trabecular separation (Tb.Sp.) (**d**) of the metaphyseal proximal region of tibias in the MDA-MB-231 mouse model in comparison to healthy age-and sex matched controls (Healthy n=8, Vehicle n=20, R992 n=16). Data are means ± SEMs. Statistical significance was determined by two-tailed unpaired t-test.

Supplementary Fig. 20: R992 increases osteoblast numbers and reduces osteoclast precursors in the H460 lung cancer bone metastasis model.

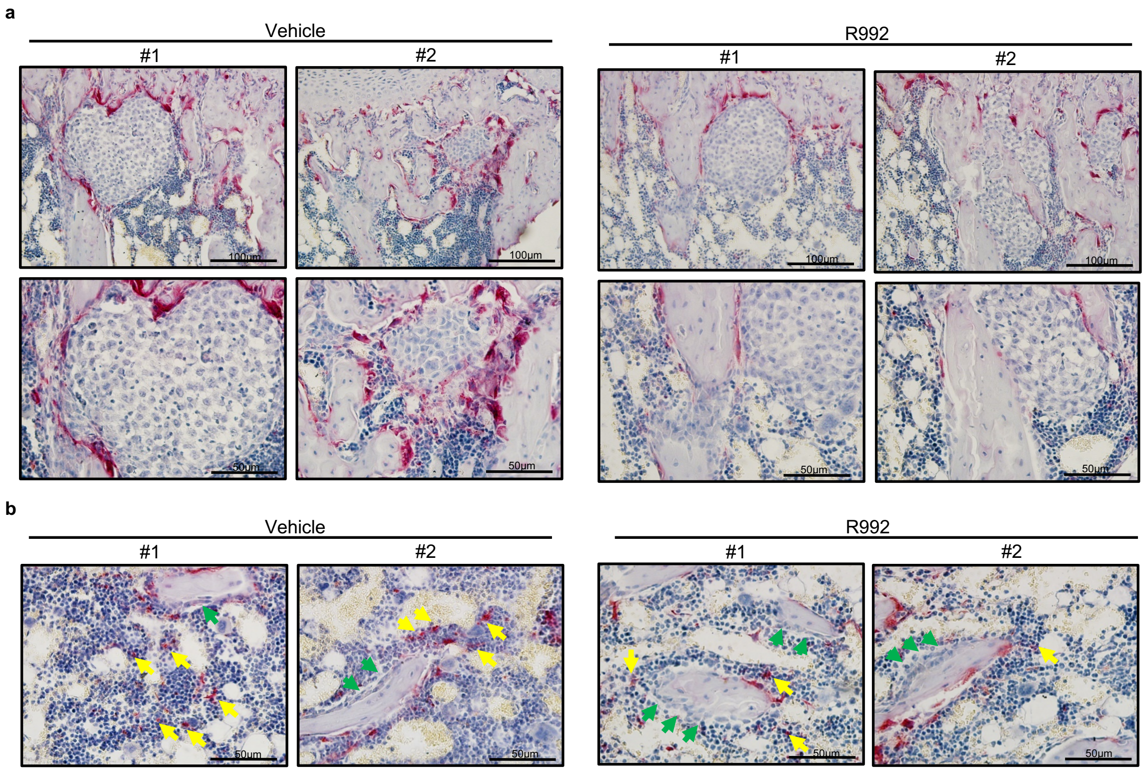

a, n=2 representative pictures of TRAP Hematoxylin staining (osteoclasts stained in red) of tibia of vehicle and R992 treated mice after intracardial injection with H460 lung cancer cells showing tumor growth in the bone with surrounding osteoclast formation. **b,** n=2 representative pictures of TRAP Hematoxylin staining showing osteoblasts visible as cuboidal or polygonal mononuclear cells on the endosteal bone surface (arrows in green) and TRAP positive mononuclear cells in the bone marrow indicating osteoclast precursor cell recruitment (arrows in yellow).

Supplementary Figure 21: Cancer cells inhibit osteoblast differentiation and function via MERTK

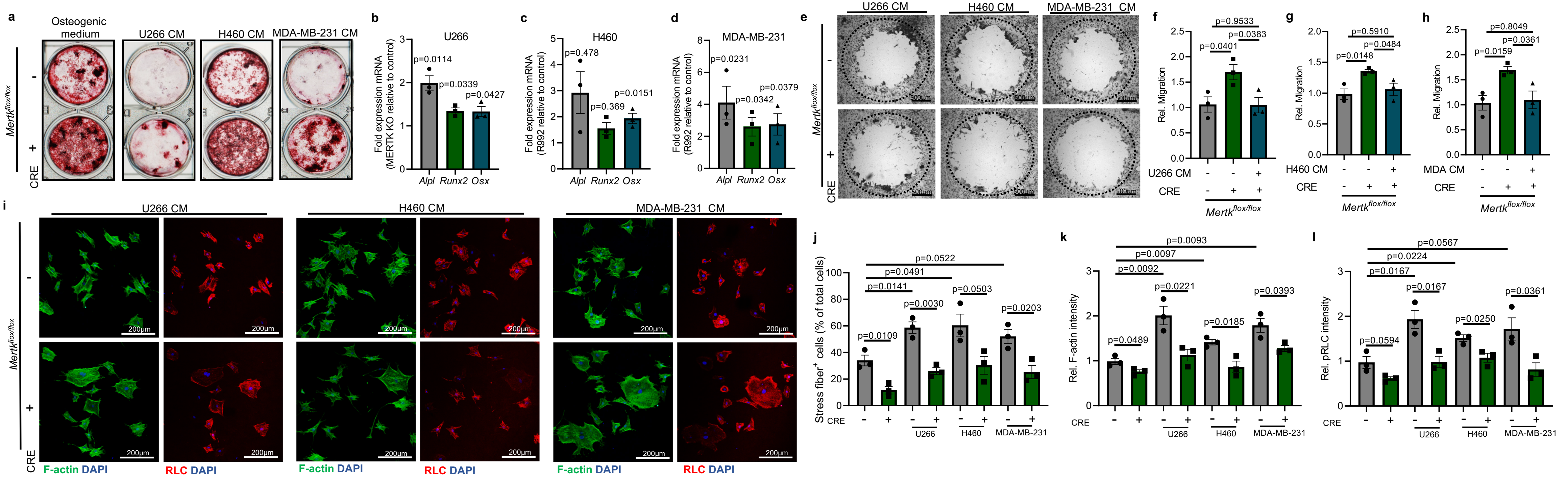

a, Photographs of Alizarin Red staining of MERTK KO osteoblast cultures incubated with osteogenic medium or tumor conditioned medium (CM) of U266, H460 and MDA-MB-231 cancer cells. **b,c,d,** Analysis of *Alpl*, *Runx2* and *SP7* mRNA expression in MERTK KO osteoblasts in the presence of tumor CM from U266 **(b)**, H460 **(c)** or MDA-MB-231 **(d)**. The bars show the relative expression compared to *Mertk$^{flox/flox}$* osteoblasts incubated with the respective tumor CM (n=3 biological replicates). **e,** Photographs of wound healing migration assays of MERTK KO osteoblast cultures incubated with tumor CM of U266, H460 and MDA-MB-231 cancer cells. **f,g,h,** Analysis of migration in MERTK KO osteoblasts induced by tumor CM from U266 **(f)**, H460 **(g)** or MDA-MB-231 **(h)** (n=3 biological replicates). **i,** Confocal imaging of F-actin and pRLC staining of MERTK KO osteoblasts on fibronectin coated glass cover slips. MERTK KO osteoblasts were incubated with tumor conditioned medium (CM) of U266, H460 and MDA-MB-231 cells for 48h. **j,** Stress fibers induced by tumor CM +/- R992 were quantified (n=100 measurements in 3 fields). **k,** F-actin immunofluorescence staining intensity was measured (n=50 measurements in 3 fields). **l,** pRLC immunofluorescence staining intensity was quantified (n=50 measurements in 3 fields). Data are means ± SEMs. Statistical significance was determined by two-tailed unpaired t-test.

Supplementary Methods: Gating strategy of myeloma plasma cell detection in myeloma mouse models.

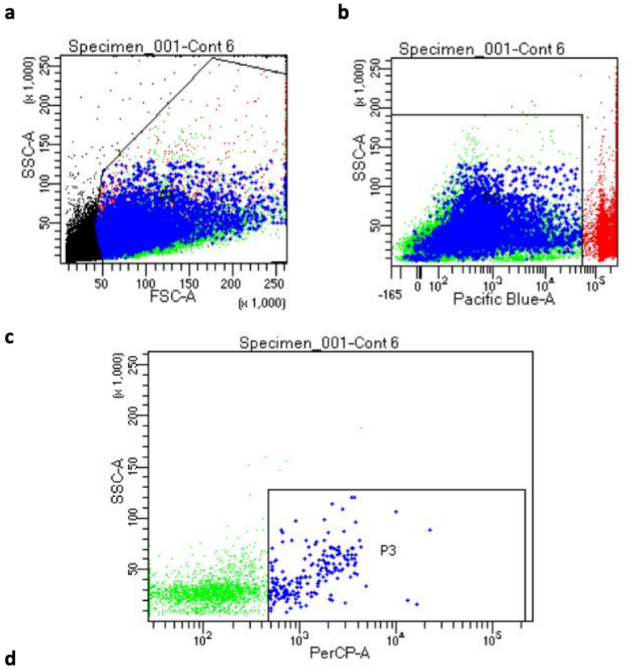

d

Tube: Cont 6

Population	#Events	%Parent	%Total
All Events	100,000	####	100.0
P1	65,928	65.9	65.9
P2	59,549	90.3	59.5
P3	3,835	6.4	3.8

a-d, Gating strategy for detection of MM cells in the bone marrow of NSG mice. As an example serves the original FACS plots of the analysis of human CD138 in a control mouse in the U266 model. In the RPMI8226 mouse model we used RPMI8226 cells transduced with a vector expressing Venus fluorescence. Cells were detected by FACS in AlexaxFluor430 channel with the same gating strategy. **a**, Forward (FSC-A) and sideward (SSC-A) scattern. **b**, DAPI was used to exclude dead cells and measured in Pacific Blue-A channel. **c,** CD138 was measured in the PerCP-A channel. **d,** Quantification of the indicated gates.

Supplementary Table: Antibodies used in this study

Antibody	Host animal	Dilution	Source	Catalog no	Application
RHOA	mouse	1:500	Cell Biolabs Inc.	STA-403-A<br>Part No. 240302	Western Blot
RHOA	rabbit	1:1000	abcam	ab86297	Western Blot
MERTK	goat	1:1000	R&D Systems	AF591	Western Blot
Phospho-MERTK	rabbit	1:1000	Phosphosolutions	p186-749	Western Blot
VAV2	rabbit	1:1000 / 1:100	Cell Signaling	#2848	Western Blot / Immunoprecipitation
Phospho-Tyrosine	mouse	1:1000	Sigma-Aldrich	05-321MG	Western Blot
TYRO3	rabbit	1:1000	Cell Signaling	#5585	Western Blot
Phospho-AKT (Ser473)	rabbit	1:1000	Cell Signaling	#4058	Western Blot
β-ACTIN	mouse	1:1000	Santa Cruz Biotechnology	sc-47778	Western Blot
Rabbit IgG (H+L) (HRP)	goat	1:10000	Novus Biologicals	NB7160	Western Blot
Mouse igG (H+L) (HRP)	rabbit	1:10000	Novus Biologicals	NB7544	Western Blot
Phospho-MLC2	rabbit	1:100	Cell Signaling	#3671	Immunofluorescence
Alexa Fluor 488 Phalloidin	n.a.	1:50	Invitrogen	A12379	Immunofluorescence
VINCULIN	rabbit	1:500	Sigma-Aldrich	V4139	Immunofluorescence
Rabbit igG (H+L) AlexaFluor 555	rabbit	1:200-1:500	Invitrogen	A-21428	Immunofluorescence
