# Supplementary material for: Regulation of bone homeostasis by MERTK and TYRO3
Source: Nat Commun. 2022 Dec 12;13:7689. doi: 10.1038/s41467-022-33938-x (PMC9744875; doi:10.1038/s41467-022-33938-x)
Supplement: Supplementary file 2 — Reporting Summary [file 41467_2022_33938_MOESM2_ESM.pdf]

nature portfolio

Corresponding author(s): Sonja Loges
Last updated by author(s): 26.08.22

Reporting Summary

Nature Portfolio wishes to improve the reproducibility of the work that we publish. This form provides structure for consistency and transparency in reporting. For further information on Nature Portfolio policies, see our Editorial Policies and the Editorial Policy Checklist.

Statistics

For all statistical analyses, confirm that the following items are present in the figure legend, table legend, main text, or Methods section.

n/a	Confirmed	
☐	☒	The exact sample size (*n*) for each experimental group/condition, given as a discrete number and unit of measurement
☐	☒	A statement on whether measurements were taken from distinct samples or whether the same sample was measured repeatedly
☐	☒	The statistical test(s) used AND whether they are one- or two-sided<br>*Only common tests should be described solely by name; describe more complex techniques in the Methods section.*
☒	☐	A description of all covariates tested
☒	☐	A description of any assumptions or corrections, such as tests of normality and adjustment for multiple comparisons
☒	☐	A full description of the statistical parameters including central tendency (e.g. means) or other basic estimates (e.g. regression coefficient) AND variation (e.g. standard deviation) or associated estimates of uncertainty (e.g. confidence intervals)
☐	☒	For null hypothesis testing, the test statistic (e.g. *F*, *t*, *r*) with confidence intervals, effect sizes, degrees of freedom and *P* value noted<br>*Give P values as exact values whenever suitable.*
☒	☐	For Bayesian analysis, information on the choice of priors and Markov chain Monte Carlo settings
☒	☐	For hierarchical and complex designs, identification of the appropriate level for tests and full reporting of outcomes
☒	☐	Estimates of effect sizes (e.g. Cohen's *d*, Pearson's *r*), indicating how they were calculated

Our web collection on statistics for biologists contains articles on many of the points above.

Software and code

Policy information about availability of computer code

Data collection	Scanco Medical Systems and Software for measurement of bone parameters.
Data analysis	GraphPad Prism 5 software, BD FACSDiva software, Osteomeasure software program (OsteoMetrics, Inc.), Living Image software (Perkin Elmer).

For manuscripts utilizing custom algorithms or software that are central to the research but not yet described in published literature, software must be made available to editors and reviewers. We strongly encourage code deposition in a community repository (e.g. GitHub). See the Nature Portfolio guidelines for submitting code & software for further information.

Data

Policy information about availability of data

All manuscripts must include a data availability statement. This statement should provide the following information, where applicable:
- Accession codes, unique identifiers, or web links for publicly available datasets
- A description of any restrictions on data availability
- For clinical datasets or third party data, please ensure that the statement adheres to our policy

The raw data generated in this study is provided in the Source Data file.

Field-specific reporting

Please select the one below that is the best fit for your research. If you are not sure, read the appropriate sections before making your selection.

- [x] Life sciences
- [ ] Behavioural & social sciences
- [ ] Ecological, evolutionary & environmental sciences

For a reference copy of the document with all sections, see nature.com/documents/nr-reporting-summary-flat.pdf

Life sciences study design

All studies must disclose on these points even when the disclosure is negative.

Sample size	The small molecule inhibitor R992 has been used before in an in vivo syngeneic, murine model of colorectal carcinoma, where R992 inhibited tumor growth with 60 mg/kg. In this model a mean tumor reduction of 30% could be observed (Holland et al., AACR, 2016). As there was no further data of the effect of R992 available, we calculated the sample size on the basis of the colorectal carcinoma model. With a potential anti-tumor activity of R992 of about 30% we calculated the Cohens d effect size with the standard deviation of maximum 20%. Our calculations showed a value of (d = 1,75; r = 0,66). Therefore we planned our experiments with a sample size of 10 animals. All sample size calculations were performed using G* Power 3.1 software.
Data exclusions	We calculated significant outliers using ESD method (extreme studentized deviate) and excluded significant outliers from the analysis. In the myeloma mouse models, we measured iglamda light chain in the peripheral blood to monitor tumor load. Animals were excluded from analysis which did not show any Iglambda concentrations in ELISA. After intracardiac injection of H460 and MDA-MB-231 cells, we monitored tumor load by bioluminescence imaging (BLI). Mice showing exclusively BLI signals in the lung or no did not show any signals were excluded from analysis
Replication	All in vitro experiments were performed at least 3 times with similar results. For the manuscript one representative result per experiment was chosen. In vivo experiments could not be repeatedly done due to animal welfare regulations but the usage of R992 in different mouse models with similar results is an indicator of reproducibility of the effects observed by R992 in vivo.
Randomization	Mice were randomly assigned to one of the treatment conditions. In all cell culture experiments cells were split and randomly assigned to untreated or treatment group.
Blinding	No blinding was performed during the application of R992 as the optical appearance of the drug was slightly different in comparison to the Placebo/Vehicle. Blinding was performed during the data collection and analysis of mouse experiments.<br>During in vitro single cell counting experiments investigators were blinded to group allocation (counting of stress fiber pos. cells, polarized cells)

Reporting for specific materials, systems and methods

We require information from authors about some types of materials, experimental systems and methods used in many studies. Here, indicate whether each material, system or method listed is relevant to your study. If you are not sure if a list item applies to your research, read the appropriate section before selecting a response.

Materials & experimental systems

n/a	Involved in the study	
[ ]	[x]	Antibodies
[ ]	[x]	Eukaryotic cell lines
[x]	[ ]	Palaeontology and archaeology
[ ]	[x]	Animals and other organisms
[x]	[ ]	Human research participants
[x]	[ ]	Clinical data
[x]	[ ]	Dual use research of concern

Methods

n/a	Involved in the study	
[x]	[ ]	ChIP-seq
[ ]	[x]	Flow cytometry
[x]	[ ]	MRI-based neuroimaging

Antibodies

Antibodies used	RHOA mouse 1:500 Cell Biolabs Inc. STA-403-A Part No. 240302 Western Blot<br>RHOA rabbit 1:1000 abcam ab86297 Western Blot<br>MERTK goat 1:1000 R&D Systems AF591 Western Blot<br>Phospho-MERTK rabbit 1:1000 Phosphosolutions p186-749 Western Blot<br>VAV2 rabbit 1:1000 / 1:100 Cell Signaling #2848 Western Blot / Immunoprecipitation<br>Phospho-Tyrosine mouse 1:1000 Sigma-Aldrich 05-321MG Western Blot<br>TYRO3 rabbit 1:1000 Cell Signaling #5585 Western Blot<br>Phospho-AKT (Ser473) rabbit 1:1000 Cell Signaling #4058 Western Blot<br>β-ACTIN mouse 1:1000 Santa Cruz sc-47778 Western Blot

	Rabbit IgG (H+L) (HRP) goat 1:10000 Novus Biologicals NB7160 Western Blot<br>Mouse igG (H+L) (HRP) rabbit 1:10000 Novus Biologicals NB7544 Western Blot<br>Phospho-MLC2 rabbit 1:100 Cell Signaling #3671 Immunofluorescence<br>Alexa Fluor 488 Phalloidin n.a. 1:50 Invitrogen A12379 Immunofluorescence<br>VINCULIN rabbit 1:500 Sigma-Aldrich V4139 Immunofluorescence<br>Rabbit igG (H+L) AlexaFluor 555 rabbit 1:200-1:500 Invitrogen A-21428 Immunofluorescence
Validation	Anti-RHOA (Cellbiolabs): Part of a RhoA activation assay. Validated for murine cells. >20 original articles are cited on the manufacturers website showing RhoA Western Blots.<br><br>Anti-RHOA (abcam): Validated for Western Blot application in murine cells stated on the manufactures website. >10 original articles shown on manufacturers website implicate RhoA Western Blots.<br><br>Anti-MERTK (R&D): Product datasheet states it detects mouse MERTK in Western Blot. >20 publications cited on manufacturers website are showing MERTK Western Blot.<br><br>Anti-phospho-MERTK (Phosphosolutions): Manufacturers website states it detects phospho-MERTK in human and murine cells in Western Blots.<br><br>Anti-VAV2 (Cell Signaling): According to manufacturers website validated for Western Blot and Immunoprecipitation. 6 original articles are cited on manufacturers website showing Western Blot applications in murine cells.<br><br>Anti-phospho-Tyrosine (Sigma Aldrich): According to manufacturers website validated and published for Western Blot applications.<br><br>Anti-TYRO3 (Cell Signaling): According to manufacturers website validated for Western Blot. >20 original articles are cited on manufacturers website showing Western Blot applications in murine cells.<br><br>Anti-phospho-Akt (Cell Signaling): According to manufacturers website validated for Western Blot. >1000 original articles are cited on manufacturers website showing Western Blot applications in murine cells.<br><br>Anti-ß-ACTIN (Santa Cruz Biotechnology): According to manufacturers website validated for Western Blot. >1000 original articles are cited on manufacturers website showing Western Blot applications in human and murine cells.<br><br>Anti-phospho-MLC2 (Cell Signaling): According to manufacturers website validated for Immunofluorescence. >100 original articles are cited on manufacturers website showing Immunofluorescence applications in murine cells.<br><br>Anti-Vinculin (Sigma Aldrich) : According to manufacturers website validated for Immunofluorescence. >10 original articles are cited on manufacturers website showing Immunofluorescence applications in murine cells.

Eukaryotic cell lines

Policy information about cell lines

Cell line source(s)	U266: DSMZ , RPMI8226: DSMZ, H460: Institute of Tumor Biology (UKE), MDA-MB-231: Institute of Tumor Biology (UKE), EO771: Institute of Tumor Biology (UKE)
Authentication	Human cell lines were frequently authenticated using Multiplex human Cell line Authentication Test (MCA) by Multiplexion. The last authentication was performed for MDA-MB-231 and U266 in 2021 and for H460 and RPMI822 in 2020 after the indicated experiments.
Mycoplasma contamination	All cell lines were frequently tested negative for mycoplasma contamination.
Commonly misidentified lines (See ICLAC register)	According to the ICLAC register no commonly misidentified cell lines were used in this study.

Animals and other organisms

Policy information about studies involving animals; ARRIVE guidelines recommended for reporting animal research

Laboratory animals	Strains:<br>NSG (NOD.C-Prkdcscidll2rgtm1Wjl/SzJ): 6-8w old females used in experiments.<br><br>B6 (C57BL/6J): 8w old females used in experiment.<br><br>Col1a1-2.3kb-Cre (C57BL/6 background): Used for breeding of conditional knockout mice.<br><br>Mertkflox/flox (C57BL/6 background): Used for breeding of conditional knockout mice.

	Tyro3flox/flox (C57BL/6 background): Used for breeding of conditional knockout mice.<br><br>Mertk-/-OB: Col1a1-2.3kb-Cre;Mertkflox/flox (C57BL/6 background): 8 week old females used in experiments.<br><br>Tyro3-/-OB: Col1a1-2.3kb-Cre;Tyro3flox/flox (C57BL/6 background): 8 week old females used in experiments.
Wild animals	no wild animals were used in this study.
Field-collected samples	no field collected samples were used in this study.
Ethics oversight	All animal experiments were carried out in concordance with the institutional guidelines for the welfare of animals in experimental neoplasia and were approved by the local licensing authority (Behörde für Soziales, Gesundheit, Familie, Verbraucherschutz; Amt für Gesundheit und Verbraucherschutz, project number G65/17, N24/19, N30/19, N119/21).

Note that full information on the approval of the study protocol must also be provided in the manuscript.

Flow Cytometry

Plots

Confirm that:

- [x] The axis labels state the marker and fluorochrome used (e.g. CD4-FITC).
- [x] The axis scales are clearly visible. Include numbers along axes only for bottom left plot of group (a 'group' is an analysis of identical markers).
- [x] All plots are contour plots with outliers or pseudocolor plots.
- [x] A numerical value for number of cells or percentage (with statistics) is provided.

Methodology

Sample preparation	Bone marrow of multiple myeloma bearing mice was flushed from the femurs. Mice were injected intrafemorally with U266 wild type or RPMI8226-Venus human MM cells. Erythrolysis was done for 10 minutes on ice.
Instrument	FACS analysis was done with BD Fortessa
Software	BD FACSDivaTM software
Cell population abundance	The abundance of the CD138 population was between 3% and 8% and was determined by PerCP-Cy5.5 fluorescence.<br>The abundance of RPMI8226-Venus cells was between 0.8% and 80% and determined by AlexaFluor430 fluorescence.
Gating strategy	SSC-A/FSC-A, with an unstained sample the boundaries were defined above 10 to the 3.

- [x] Tick this box to confirm that a figure exemplifying the gating strategy is provided in the Supplementary Information.
